# Chemical compositions, chromatographic fingerprints and antioxidant activities of Citri Exocarpium Rubrum (*Juhong*)

**DOI:** 10.1186/s13020-017-0127-z

**Published:** 2017-01-25

**Authors:** Yang Zhao, Chun-Pin Kao, Chi-Ren Liao, Kun-Chang Wu, Xin Zhou, Yu-Ling Ho, Yuan-Shiun Chang

**Affiliations:** 1 Department of Chinese Pharmaceutical Sciences and Chinese Medicine Resources, College of Biopharmaceutical and Food Sciences, China Medical University, Taichung, 40402 Taiwan; 2 Key Laboratory for Information System of Mountainous Areas and Protection of Ecological Environment, Guizhou Normal University, Guiyang, 550001 China; 3 Department of Nursing, Hsin Sheng College of Medical Care and Management, Taoyuan, 32544 Taiwan; 4 Department of Nursing, Hungkuang University, Taichung, 43302 Taiwan; 5 Chinese Crude Drug Pharmacy, China Medical University Hospital, Taichung, 40402 Taiwan

## Abstract

**Background:**

Citri Exocarpium Rubrum (CER), which is known as *Juhong* in Chinese, is the dried exocarp of *Citrus reticulata* Blanco and its cultivars (Fam. Rutaceae) and is currently used in Chinese medicine to protect the stomach and eliminate *dampness* and phlegm. The main aim of this study was to develop a high-performance liquid chromatography ultraviolet mass spectrometry (HPLC-UV-MS) method for determining the chemical compositions and fingerprint of CER. We also evaluated the antioxidant properties of CER based on its 2,2-diphenyl-1-picrylhydrazyl (DPPH) free radical scavenging activity, ferric ion reducing antioxidant power (FRAP) and trolox equivalent antioxidant capacity (TEAC) assays.

**Methods:**

Ten CER samples were collected from Hong Kong and mainland China. Each CER sample was extracted using an ultrasonic extraction method. Chromatographic separation was achieved using a conventional Dikma Inspire C18 column with photo diode array detection (190–400 nm). Hesperidin, nobiletin and tangeretin were quantified based on the UV signal observed at 330 nm. The column was eluted with a mobile phase consisting of water and acetonitrile (15–55%) over 55 min. Fingerprints combined with similarity and principal component analyses were used to classify the herbs. The DPPH free radical scavenging activity, FRAP and ABTS properties of the different CER samples were assayed. Bivariate correlation analysis was performed to investigate the correlation between the characteristic peaks and their antioxidant capacities.

**Results:**

Limit of detection (LOD), limit of quantification (LOQ), linearity, inter-day precision, intra-day precision, repeatability, stability and recovery of the developed method were validated, and the method was subsequently used to determine the contents of hesperidin, nobiletin and tangeretin, and to acquire the fingerprints of the CER samples. Seventeen characteristic peaks were found in the fingerprints, and eleven of them were identified. Bivariate correlation analysis revealed correlations between the characteristic peaks and the antioxidant activities of the samples.

**Conclusion:**

An HPLC-UV-MS method was developed and validated after a detailed investigation on extraction of chemical compounds from CER using different solvents and extraction times. None of the peaks was correlated with the DPPH free radical scavenging activity or ferric reducing capacity. Most of the peaks were correlated well with the ABTS radical scavenging capacity.

**Electronic supplementary material:**

The online version of this article (doi:10.1186/s13020-017-0127-z) contains supplementary material, which is available to authorized users.

## Background

Citri Exocarpium Rubrum (CER), which is known as *Juhong* in Chinese, is the dried exocarp of *Citrus reticulata* Blanco and its cultivars (Fam. Rutaceae), and this material is used in Chinese medicine to protect the stomach and eliminate *dampness* and phlegm [1]. CER has been reported to exhibit antioxidant activity [2] and induce apoptosis in human lung cancer cells [3]. Hesperidin, nobiletin and tangeretin are the main bioactive components of CER, and these compounds have been reported to have several properties, e.g. including antioxidant [4], anti-inflammatory [5] and anticancer activities [5, 6]. However, hesperidin is the only one of these compounds currently described as a chemical marker for quality assessment of CER in the 2015 edition of the Chinese Pharmacopoeia [1], which stipulates that the content of the marker should not be less than 1.7%. Several quantitative quality control methods have been reported for CER and CER-related commercial products [7–9]. However, the quality of herbal materials cannot be thoroughly or accurately evaluated based on the quantification of one or two chemical components. Fingerprinting analysis, which represents a much more accurate form of quality control analysis has therefore been introduced and accepted by the World Health Organization (WHO) as an effective strategy for assessing the quality of herbal medicines [10–13].

The aim of this study was to develop a high-performance liquid chromatography ultraviolet mass spectrometry (HPLC-UV-MS) method to determine the hesperidin, nobiletin and tangeretin contents in different CER samples, as well as developing a deeper understanding of the chemical profiles of these materials. Similarity and principal component analyses (PCA) were also performed based on the contents of hesperidin, nobiletin and tangeretin, as well as the PA/W (peak area divided by sample weight) values of the 17 characteristic peaks. The antioxidant activities of the CER samples were evaluated and subjected to a fingerprint-efficacy correlation process.

## Methods

### Chemicals, solvents and herbal materials

Hesperidin, nobiletin and tangeretin were purchased from Shanghai R & D Center for Standardization of Traditional Chinese Medicines (China). LC-grade methanol and acetonitrile were purchased from the branch company of Merck in Taipei, Taiwan. Purified water was prepared with Milli-Q system (Millipore, Milford, MA, USA). All other reagents used in the present study were of analytical grade. Ten batches of CER herbal materials were collected from Hong Kong and mainland China, which were marked as L-CER-01–L-CER-04 (from local in Hong Kong) and CER-01–CER-06 (from mainland China). All the samples were authenticated by professor Yuan-Shiun Chang and the specimens have been deposited in Department of Chinese Pharmaceutical Sciences and Chinese Medicine Resources, School of Pharmacy, China Medical University.

### Preparation of herbal materials and reference compounds

CER samples were dried in a shade place and were ground into fine powder (20 mesh) using a grinder with a knife blade. Two hundred milligrams of each CER powder was carefully weighed into a tube. Methanol (20 mL) was then added into the tube. Each sample was then extracted using an ultrasonic cleaner (Delta DC400H) at a frequency of 40 kHz at 25 °C for 30 min. The extract was centrifuged (UX-17414-21 Cole-Parmer™ MS-3400 Variable Speed Clinical Centrifuge, USA) for 10 min at 2000×*g* and the supernatant was then transferred into a volumetric flask. The procedure was repeated for one more time and the supernatants were combined. The final volume was made up to 50 mL with methanol which was then filtered through a 0.45 μm PVDF syringe filter (VWR Scientific, Seattle, WA) for analysis.

The reference compounds of hesperidin, nobiletin and tangeretin were weighed and dissolved in methanol at 499.2, 11.94 and 5.0288 mg/L (stock solutions), respectively. The stock solutions were then diluted to 31.20–499.20 mg/L for hesperidin, 0.75–11.94 mg/L for nobiletin and 0.31–5.03 mg/L for tangeretin, respectively for establishment of calibration curves. An aliquot of 10 μL of each solution was used for HPLC and HPLC-ESI-MS analyses.

### HPLC analysis

HPLC analyses were performed on a Waters 2695 HPLC system (Waters Corporation, USA) equipped with Waters 2998 photodiode array detector (PDA), Waters e2695 separations module and column heater module. A Dikma Inspire, C18 column (250 mm × 4.6 mm i.d., 5 μm) was used. The mobile phase consisted of water (A) and acetonitrile (B). The optimized elution conditions were as follow: 0–10 min, 15–20% B; 10–25 min, 20% B; 25–35 min, 20–40%; 35–55 min, 40–55%. The flow rate was 1 mL/min and the injection volume was 10 μL. UV spectra were acquired from 190 to 400 nm. The autosampler and column compartment were maintained at 25 and 35 °C, respectively.

### HPLC-ESI-MS analysis

HPLC-ESI-MS analyses were performed on a TSQ Quantum Access Max Triple Stage Quadrupole Mass Spectrometer (Thermo Fisher Scientific Inc., Waltham, MA, USA) with an Accela 1250 UHPLC system equipped with an Accela 1250 PDA detector, an Accela HTC PAL autosampler, and an Accela 1250 binary pump. The column and elution conditions used were the same as that described in the section, “HPLC analysis”, except that the flow rate was set at 0.20 mL/min with a split ratio. Ultrahigh pure helium (He) and high purity nitrogen (N_2_) were used as collision gas and nebulizer, respectively. The optimized parameters in negative/positive ion modes were as follows: ion spray voltage, −2.5 kV/3.5 kV; auxiliary gas, 40 arbitrary units; sheath gas, 15 arbitrary units; capillary temperature, 350 °C; vaporizer temperature, 300 °C; capillary offset, −30 V/30 V. Spectra were recorded in the range of *m/z* 100–1500 for full scan data, meanwhile, the normalized collision energy was tested from 25 to 45% for MS^2^ data with dependent scan.

### Antioxidant activities

#### DPPH assay

The DPPH free radical-scavenging activity of each sample was assayed according to published articles [14–16] with minor modifications. In brief, a 0.3 mM solution of methanolic DPPH was freshly prepared. An aliquot (50 μL) of each sample (with appropriate enrichment or dilution if necessary) was added to 150 μL of methanolic DPPH solution to initiate the reaction. Discolorations were measured at 517 nm after reaction for 30 min at room temperature in the dark. Measurements were performed in triplicate. The %DPPH quenched was calculated according to the following equation:

$${\text{\% DPPH quenched}} = {{( {{\text{A}}_{{{\text{control}}}} \,- \,{\text{A}}_{{{\text{sample}}}} })  \times 100} / {{\text{A}}_{{{\text{control}}}} \, - \,{\text{A}}_{{{\text{blank}}}} }}$$

where A_control_, A_sample_, and A_blank_ represent the absorbance of the control, the selected antioxidant sample at certain concentration, and the blank, respectively, measured at 517 nm. EC_50_ values calculated denote the concentration of a sample required to decrease the absorbance by 50%. The antioxidant capacity was expressed as μmol butylated hydroxytoluene (BHT) equivalents/gram of dried CER sample.

#### FRAP assay

The ability to reduce ferric ions was measured based on the methods reported in previous papers [16, 17] with minor revisions. Briefly, 30 μL of each sample (with appropriate enrichment or dilution if necessary) was added to 200 μL of FRAP reagent (10 parts of 300 mM sodium acetate buffer at pH 3.6, 1 part of 10.0 mM 2,4,6-tripyridyl-s-triazine (TPTZ) solution, and 1 part of 20.0 mM FeCl_3_·6H_2_O solution), and the reaction mixture was placed at room temperature for 5 min. Fresh working solutions of FeSO_4_·7H_2_O were used for calibration. The antioxidant capacity of reducing ferric ions of each sample was expressed as μ mol Fe^2+^ equivalents per gram of dried CER sample.

#### TEAC assay

The ABTS free radical-scavenging activity of each sample was assayed according to the method described in published papers [16, 18] with minor modifications. A mixture (1:1, v/v) of ABTS (7.0 mM) and potassium persulfate (6 mM) was allowed to react overnight at room temperature in the dark to form radical cation ABTS^+^. The mixture was then diluted with water to reach absorbance values between l.0 and 1.5 at 731 nm (constant initial absorbance values must be used for standard and samples) as working solution. An aliquot (50 μL) of each sample (with appropriate enrichment or dilution if necessary) was mixed with the working solution (150 μL), and the decrease of absorbance was measured at 731 nm after 10 min. EC_50_ values calculated denote the concentration of a sample required to decrease the absorbance by 50%. The antioxidant capacity was expressed as μmol BHT equivalents/gram of dried CER sample.

### Data analysis

#### Similarity analysis

Similarity values of the chromatographic fingerprints obtained from CER samples were calculated using the following two formulas:

$$ {\text{Correlation coefficient}}\; = \;\frac{{\sum\nolimits_{i = 1}^{n} {(X_{i} { - }\bar{X}_{i} )(Y_{i} - \bar{Y}_{i} )} }}{{\sqrt {\sum\nolimits_{i = 1}^{n} {(X_{i} - \bar{X}_{i} )^{2} (Y_{i} { - }\bar{Y}_{i} )^{2} } } }} $$

$$ {\text{Angle cosin}}\; = \;\frac{{\sum\nolimits_{i = 1}^{n} {X_{i} Y_{i} } }}{{\sqrt {\sum\nolimits_{i = 1}^{n} {X_{i}^{2} } } \sqrt {\sum\nolimits_{i = 1}^{n} {Y_{i}^{2} } } }} $$

where, X_i_ and Y_i_ represented the peak area of the characteristic peak in each sample and the peak area of the characteristic peak in reference fingerprint generated, respectively.

#### Principal component analysis (PCA)

The data obtained from chromatographic fingerprints were analyzed with Solo (Eigenvector Research, Inc.,Wenatchee, WA). Normalize (2-Norm, length = 1) and mean center were used for data reprocessing before PCA was performed.

#### One-way ANOVA and correlation analyses

Differences in mean values of antioxidant assays for different CER samples were obtained by one-way ANOVA. Pearson r correlation coefficients between the peak areas of the seventeen characteristic peaks and their antioxidant capacities were calculated by SPSS software (version 17.0, IBM Corporation, USA). *P* values less than 0.05 were considered statistically significant.

## Results and discussion

### Optimization of extraction method

The extraction solvents were optimized taking the extraction efficiency of hesperidin, nobiletin and tangeretin as indexes. Methanol, 50% methanol, ethanol and 50% ethanol were investigated with ultrasonic extraction at room temperature. The powder of CER (0.2 g) was extracted with 20 mL of different solvents for twice (30 min for once). As a result, the contents of the three analytes obtained by methanol were the highest compared with the values obtained from other solvents. Furthermore, the three analytes were almost extracted completely (>99%) for the second time (Additional files 1 and 2).

### Optimization of chromatographic conditions

**Fig. 1:**
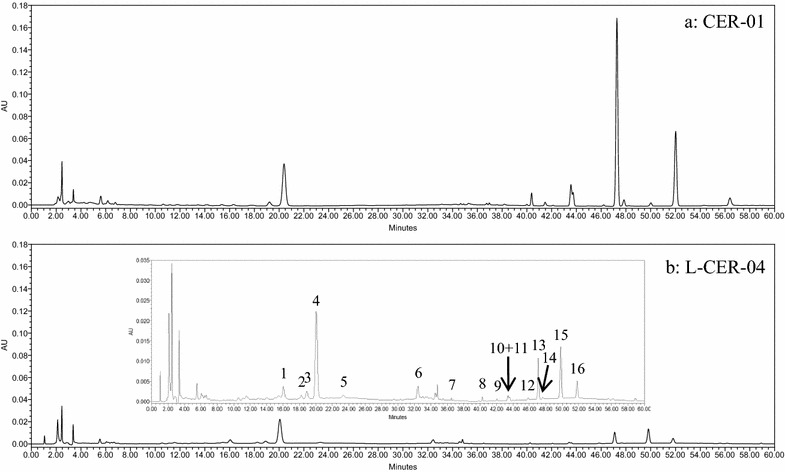
Representative chromatograms of CER-01 (**a**) and CER-04 (**b**) detected at 330 nm. Seventeen characteristic peaks are marked as 1–17

Different mobile phase systems, methanol–water and acetonitrile–water elution systems were tested to obtain sharp and symmetrical peaks. Good resolution, baseline, sharp and symmetrical peaks were obtained by acetonitrile–water system. Two other columns (Grace Alltima C18 and Waters XBridge Shield RP18) were screened before Dikma Inspire, C18 column (250 mm × 4.6 mm i.d., 5 μm) was finally selected as the column of choice (Additional file 3). PDA full scan (190–400 nm) was used to acquire all the main peaks and finally 330 nm was selected as detection wavelength. Representative chromatographic fingerprints obtained from CER-01 and L-CER-04 were shown in Fig. 1, in which characteristic peaks were marked as 1–17. Different column temperatures at 20, 25, 30 and 35 °C were also investigated. Although chromatograms detected at different temperatures didn’t show obvious differences, 35 °C was selected as the preferable one to minimize the influences from fluctuating room temperature on the chromatograms. In the process of gradient optimization, gradient time, gradient procedure and initial composition of the mobile phase were taken into consideration. Eventually, the gradient procedure was finalized, as described in “HPLC analysis” section.

### Validation of quantitative analytical method

The HPLC method was validated by defining its limit of detection (LOD), limit of quantification (LOQ), linearity, inter-day precision, intra-day precision, repeatability, stability and recovery characteristics.

Calibration curves were plotted based on a linear regression analysis of the integrated peak areas (*y*) versus the concentrations (*x*, mg/L) of hesperidin, nobiletin and tangeretin at five different levels. The LOD and LOQ values were determined amongst the baseline noise for all three of the analytes under the optimized chromatographic conditions according to the International Union of Pure and Applied Chemistry (IUPAC) definition. The LOD values were determined as the concentration of each analyte required to generate a signal with a single-to-noise (S/N) ratio of 3:1, whereas the LOQ was defined as the concentration of each analyte required to generate a signal with an S/N ratio of 10:1. The regression equations, correlation coefficients, linear ranges, LODs and LOQs for hesperidin, nobiletin and tangeretin are shown in Table 1. The correlation coefficients (*R* ^*2*^) of hesperidin, nobiletin and tangeretin were greater than 0.99, indicating good linearity.

**Table 1:** Regression data, LODs and LOQs for the three analytes assayed in HPLC-UV chromatograms

Analyte	Calibration curve^a^	*R* ^2^	Linear range (mg/L)	LOD^b^ (ng/mL)	LOQ^c^ (ng/mL)
1	*y* = 3.0149*x* *−* 21.812	0.9994	31.20–499.20	418.8	128.6
2	*y* = 41.396*x* *−* 4.1898	0.9995	0.75–11.94	6.8	24.7
3	*y* = 50.822*x* + 3.2433	0.9996	0.31–5.03	12.0	40.8

*1* Hesperidin, *2* Nobiletin, *3* Tangeretin

^a^ *y* is the peak area in UV chromatograms detected in 330 nm, *x* is the concentration (mg/L) of the analyte

^b^LOD refers to the limit of detection, s/n = 3

^c^LOQ refers to the limit of quantification, s/n = 10

Intra- and inter-day variations were chosen to determine the precision of our newly developed method. For the intra-day variability test, one of the mixed standard solutions (hesperidin, 124.80 mg/mL; nobiletin, 2.99 mg/mL; tangeretin, 1.26 mg/mL) was analyzed five times within 1 day, whereas one of the mixed standard solutions was examined in triplicate on a daily basis for three consecutive days for the inter-day variability test. Repeatability was evaluated by analyzing five different working solutions prepared from the same sample (L-CER-01). The stability of the method was determined by the repeated analysis of the same sample solution at different times during a 24 h period of storage at room temperature. The RSD values for the retention times and peak areas of hesperidin, nobiletin and tangeretin were taken as the measurements of precision and stability. The RSDs of the retention times and contents (mg/g) were taken as the measurements of repeatability. All of the RSD values for the retention times and peak areas (or contents) were found to be less than 2.00 and 3.00%, respectively (Table 2).

**Table 2:** Results of precision, repeatability and stability of the three analytes, expressed as RSD (%)

Analyte	Precision				Repeatability (n = 5)		Stability (n = 6)	
	Intra-day RSD (%) (n = 5)		Inter-day RSD (%) (n = 9)		RSD (%)		RSD (%)	
	t_R_^a^	PA^b^	t_R_	PA	t_R_	Contents (mg/g)	t_R_	PA
1	0.32	0.39	1.39	2.98	0.52	0.94	1.08	1.65
2	0.42	0.44	1.76	1.31	0.07	1.08	0.72	2.71
3	0.47	0.49	1.65	1.69	0.07	0.88	1.53	1.34

*1* Hesperidin, *2* Nobiletin, *3* Tangeretin

^a^Refers to retention time

^b^Refers to peak area

The recovery of our newly developed method was determined using spiked CER samples. A small portion (0.2 g) of L-CER-04 was individually spiked with 7.0056 mg of hesperidin, 0.1393 mg of nobiletin and 0.0467 mg of tangeretin. Five replicate samples were extracted and analyzed according to the procedures described above. As shown in Table 3, the mean recoveries (n = 5) of hesperidin, nobiletin and tangeretin were 104.92% ± 0.67, 95.87 ± 0.72 and 96.58 ± 2.08, respectively.

**Table 3:** Recoveries of the three analytes using L-CER-04 as tested sample

Analytes	Sample weight (g)	Original (mg)	Added (mg)	Found (mg)	Recovery (%)	Mean recovery ± SD
1	0.2029	6.9687	7.0056	14.3330	105.12	104.92 ± 0.67
	0.2014	6.9172	7.0056	14.3097	105.52	
	0.2023	6.9481	7.0056	14.2179	103.77	
	0.2013	6.9137	7.0056	14.2663	104.95	
	0.2065	7.0923	7.0056	14.4638	105.22	
2	0.2029	0.1416	0.1393	0.2745	95.41	95.87 ± 0.72
	0.2014	0.1405	0.1393	0.2738	95.71	
	0.2023	0.1411	0.1393	0.2748	95.97	
	0.2013	0.1404	0.1393	0.2756	97.06	
	0.2065	0.1441	0.1393	0.2767	95.22	
3	0.2029	0.0557	0.0467	0.1009	96.91	
	0.2014	0.0556	0.0467	0.1008	96.65	
	0.2023	0.0547	0.0467	0.1006	98.14	96.58 ± 2.08
	0.2013	0.0546	0.0467	0.1005	98.13	
	0.2065	0.0568	0.0467	0.1003	93.06	

*1* Hesperidin, *2* Nobiletin, *3* Tangeretin

### Validation of the chromatographic fingerprinting method

The chromatographic fingerprinting method was validated for its inter- and intra-day precisions, repeatability and stability. The inter-day precision was evaluated by running five consecutive injections of the same test sample within 1 day. The intra-day precision was evaluated by analyzing the same test sample in triplicate each day for 3 consecutive days. The repeatability of the method was examined by determining five different solutions prepared from the same botanical sample (L-CER-04). The stability was examined by analyzing the same sample solution at different time points (0, 2, 4, 8, 16 and 24 h). The results of these experiments expressed in two forms, including (1) the RSDs of the relative retention times (RRT) and relative peak areas (RPA) of each characteristic peak relative to the reference peak (nobiletin); and (2) the similarity values for the chromatographic fingerprints of the different samples relative to the reference values, which were calculated using the formulas discussed in the “Data analysis” section. The RSD values were all less than 5%, whereas the similarity values were all greater than 0.99, indicating that our newly developed chromatographic fingerprinting method is satisfactory for the analysis of the samples.

### Quantitative determination of hesperidin, nobiletin and tangeretin contents of the CER samples

Our newly developed HPLC-UV method was used to quantify the different amounts of hesperidin, nobiletin and tangeretin in several different batches of CER. Calibration curves were generated for these compounds using reference compound and used to calculate the hesperidin, nobiletin and tangeretin contents in different samples (Table 4).

**Table 4:** Contents (mg/g dry weight, mean ± SD) of hesperidin, nobiletin and tangeretin in different CER samples

Sample no.	Content (mg/g dry weight) (Mean ± SD)			Moisture content (%)
	Hesperidin	Nobiletin	Tangeretin	
L-CER-01	23.7940 ± 0.4782	0.9882 ± 0.0047	0.5086 ± 0.0034	11.86
L-CER-02	48.9356 ± 0.1245	0.8221 ± 0.0121	0.3308 ± 0.0024	11.99
L-CER-03	39.3195 ± 0.2017	0.5771 ± 0.0109	0.2155 ± 0.0055	13.39
L-CER-04	42.6070 ± 0.5382	0.7187 ± 0.0109	0.3069 ± 0.0049	8.14
CER-01	79.5846 ± 0.5380	14.6451 ± 0.0889	5.6522 ± 0.0329	9.33
CER-02	73.2414 ± 0.5380	14.6177 ± 0.6494	5.5453 ± 0.2511	9.60
CER-03	71.6286 ± 0.0907	14.9612 ± 0.0022	5.7574 ± 0.0011	9.20
CER-04	73.7426 ± 0.2926	15.6881 ± 0.0637	6.0309 ± 0.0244	8.39
CER-05	41.2002 ± 0.2510	2.3286 ± 0.0193	1.2122 ± 0.0103	7.96
CER-06	39.7620 ± 0.0302	6.2006 ± 0.0090	4.0030 ± 0.0051	11.58

The contents of hesperidin, nobiletin and tangeretin in different samples varied from 23.8 to 79.6 mg/g, 0.587 to 15.7 mg/g and 0.222 to 6.03 mg/g, respectively. The differences in contents of hesperidin in different CER samples were small, whereas the differences in contents of nobiletin and tangeretin changed considerably.

All of the CER samples collected from mainland China were found to have higher hesperidin, nobiletin and tangeretin contents than those collected locally from Hong Kong. CER-01 had the highest content of hesperidin between all the samples tested in the current study at 79.6 mg/g, whereas CER-04 had the highest contents of nobiletin and tangeretin at 15.7 and 6.03 mg/g, respectively. The lowest content of hesperidin was found in L-CER-01 at 23.8 mg/g, whereas the lowest contents of nobiletin and tangeretin were both found in L-CER-03 at 0.588 and 0.222 mg/g, respectively.

### Assignment of the characteristic peaks

Figure 1 shows the 17 characteristic peaks detected at 330 nm in the CER-01 and L-CER-04 samples. The structures responsible for these peaks were identified based on their MS and MS^2^ behaviors and a comparison of these data with those in the literature (Table 5). The MS conditions were optimized and the data were collected in the negative and positive ESI modes.

**Table 5:** Assignment of the characteristic peaks

No.	RT (min)	UV (nm)	MS in neg. mode	MS in pos. mode	MS^2^ in neg. mode	MS^2^ in pos. mode	Assignment	References
1	15.2	283	/	/	/	/	Unknown	/
2	18.3	282	/	/	/	/	Naringin	/
3	19.2	253, 347	607 [M–H]^−^	609 [M+H]^+^	299 [M-Rutinosyl–H]^−^	301 [M-Rutinosyl+H]^+^	Unknown	/
				631 [M+Na]^+^				
				463 [M-Rhamnosyl+H]^+^				
				301 [M-Rutinosyl+H]^+^				
4	20.1	283	609 [M–H]^−^	611 [M+H]^+^	301 [M-Rutinosyl–H]^−^	303 [M-Rutinosyl+H]^+^	Hesperidin	[7, 19]
			1219 [2M–H]^−^	1243 [2M+Na]^+^	286 [M-Rutinosyl–CH_3_–H]^−^	285 [M-Rutinosyl-H_2_O+H]^+^		
				465 [M-Rhamnosyl+H]^+^	257 [M-Rutinosyl–CO_2_–H]^−^	270 [M-Rutinosyl–H_2_O–CH_3_+H]^+^		
				303 [M-Rutinosyl+H]^+^	242 [M-Rutinosyl-CO_2_-CH_3_-H]^−^	195 [M-Rutinosyl–C_6_H_4_O_2_+H]^+^		
					134 [M-Rutinosyl-CO_2_–CH_3_–C_6_H_4_O_2_–H]^−^	177 [M-Rutinosyl–C_6_H_4_O_2_–H_2_O+H]^+^		
5	23.3	283	609 [M–H]^−^	611 [M+H]^+^	/	/	Neohesperidin	/
				633 [M+Na]^+^				
				465 [M-Rhamnosyl+H]^+^				
				303 [M-Rutinosyl+H]^+^				
6	32.4	254, 347	723 [M–H]^−^	725 [M+H]^+^	417 [M-306–H]^−^	419 [M-306+H]^+^	Unknown	/
			695 [M–CO–H]^−^	747 [M+Na]^+^				
7	36.5	210, 257, 342	/	/	/	/	Unknown	/
8	40.3	203, 269, 345	/	373 [M+H]^+^	/	343 [M–CH_2_O+H]^+^	Unknown	/
				395 [M+Na]^+^				
				358 [M–CH_3_+H]^+^				
				343 [M–CH_2_O+H]^+^				
9	42.2	206, 253, 270, 355	/	403 [M+H]^+^	/	373 [M–CH_2_O+H]^+^	Unknown	/
				425 [M+Na]^+^				
				388 [M–CH_3_+H]^+^				
				373 [M–CH_2_O+H]^+^				
10	43.6	214, 241, 330	/	373 [M+H]^+^	/	343 [M–CH_2_O+H]^+^	Sinensetin	[20, 21]
				395 [M+Na]^+^		312 [M–H_2_O–CO–CH_3_+H]^+^		
				767 [2M+Na]^+^		297 [M–H_2_O–CO–CH_2_O+H]^+^		
				358 [M–CH_3_+H]^+^				
				343 [M–CH_2_O+H]^+^				
				312 [M–H_2_O–CO–CH_3_+H]^+^				
11	43.7	270, 295, 341	/	343 [M+H]^+^	/	313 [M–CH_2_O+H]^+^	Tetramethyl-O-isoscutellarein	[20]
				707 [2M+Na]^+^		285 [M–CH_2_O–CO+H]^+^		
				328 [M–CH_3_+H]^+^				
				313 [M–CH_2_O+H]^+^				
				285 [M–CH_2_O–CO+H]^+^				
12	46.3	208, 336	/	403 [M+H]^+^	/	388 [M–CH_3_+H]^+^	Hexamethoxyflavone	[21]
				373 [M–CH_2_O+H]^+^		373 [M–CH_2_O+H]^+^		
						355 [M–CH_2_O–H_2_O+H]^+^		
						327 [M–CH_2_O–H_2_O–CO+H]^+^		
13	47.1	333	/	403 [M+H]^+^	/	388 [M–CH_3_+H]^+^	Nobiletin	[7, 20, 22]
				388 [M-CH_3_+H]^+^		373 [M–CH_2_O+H]^+^		
				373 [M-CH_2_O+H]^+^		343 [M–2CH_2_O+H]^+^		
						313 [M–3CH_2_O+H]^+^		
						283 [M–4CH_2_O+H]^+^		
						239 [M–C_10_H_12_O_2_+H]^+^		
						211 [M–C_10_H_12_O_2_–CO+H]^+^		
14	47.7	266, 322	/	343 [M+H]^+^	/	313 [M–CH_2_O+H]^+^	Tetramethyl-O-scutellarein	[20]
				365 [M+Na]^+^		299 [M–CO_2_+H]^+^		
				328 [M-CH_3_+H]^+^				
				313 [M-CH_2_O+H]^+^				
15	50.1	253, 343	/	433 [M+H]^+^	/	403 [M–CH_2_O+H]^+^	3,5,6,7,8,3′,4′-Heptamethoxyflavone	[7, 20]
				455 [M+Na]^+^		373 [M–2CH_2_O+H]^+^		
				496 [M+Na+CH_3_CN]^+^		345 [M–2CH_2_O–CO+H]^+^		
				418 [M-CH_3_+H]^+^				
				403 [M-CH_2_O+H]^+^				
16	51.8	270, 323	/	373 [M+H]^+^	/	343 [M–CH_2_O+H]^+^	Tangeretin	[7, 20, 22]
				395 [M+Na]^+^		283 [M–3CH_2_O+H]^+^		
				358 [M-CH_3_+H]^+^		312 [M–CH_3_–H_2_O–CO+H]^+^		
17	56.5	203, 254, 282, 341	/	389 [M+H]^+^	/	374 [M–CH_3_+H]^+^	5-Hydroxy-6,7,8,3′,4′-pentamethoxyflavone	[7]
				411 [M+Na]^+^		359 [M–CH_2_O+H]^+^		
				374 [M-CH_3_+H]^+^				
				359 [M-CH_2_O+H]^+^				

Peak 2 had a retention time of 18.3 min and a UV absorption maximum of 282 nm. Although this compound did not give intense mass ions in the negative or positive ionization mode, it was unequivocally identified as naringin based on a comparison of its absorption wavelength and retention time data with those of the reference compound. The identity of this peak was further confirmed by the addition of a standard solution of naringin to the CER extract.

Peak 4 had a retention time of 20.1 min and a UV absorption maximum at 283 nm. MS analysis in the negative ionization mode revealed [M–H]^–^ and [2M–H]^–^ ions with an *m/z* values of 609 and 1219. The fragmentation pattern of the former of these two ions was studied by MS^2^ analysis, which revealed a series of product ions with *m/z* values of 301 [M-Rutinosyl–H]^–^, 286 [M-Rutinosyl–CH_3_–H]^–^, 257 [M-Rutinosyl–CO_2_–H]^–^, 242 [M-Rutinosyl–CO_2_–CH_3_–H]^–^ and 134 [M-Rutinosyl–CO_2_–CH_3_–C_6_H_4_O_2_–H]^–^. MS analysis of peak 4 in the positive ionization mode revealed a series of protonated molecular ion peaks with *m/z* values of 611 [M+H]^+^, 1243 [2M+Na]^+^, 465 [M-Rhamnosyl+H]^+^ and 303 [M-Rutinosyl+H]^+^. The MS^2^ fragmentation of the first of these four peaks gave six fragment ions with *m/z* values of 303 [M-Rutinosyl+H]^+^, 285 [M-Rutinosyl–H_2_O+H]^+^, 270 [M-Rutinosyl–H_2_O–CH_3_+H]^+^, 195 [M-Rutinosyl–C_6_H_4_O_2_+H]^+^ and 177 [M-Rutinosyl–C_6_H_4_O_2_–H_2_O+H]^+^. This peak was unequivocally identified as hesperidin based on a comparison of its MS data with the data from the reference compound and the literature [7, 19].

Peak 5 had a retention time of 23.3 min and a UV absorption maximum of 283 nm. MS analysis in the negative ionization mode gave a peak with an *m/z* value of 609 [M–H]^–^. In contrast, analysis in the positive ionization mode gave four peaks with *m/z* values of 611 [M+H]^+^, 633 [M+Na]^+^, 465 [M-Rhamnosyl+H]^+^ and 303 [M-Rutinosyl+H]^+^. The peak was identified as neohesperidin.

Peak 10 gave a retention time of 43.6 min with UV absorption maxima at 214, 241 and 330 nm. MS analysis in the negative ion mode did not give a major mass ion signal. In contrast, MS analysis in the positive ion mode revealed several mass ions with *m/z* values of 373 [M+H]^+^, 395 [M+Na]^+^, 767 [2M+Na]^+^, 358 [M–CH_3_+H]^+^, 343 [M–CH_2_O+H]^+^ and 312 [M–H_2_O–CO–CH_3_+H]^+^. The first of these peaks was subjected to MS^2^ fragmentation analysis, which resulted in three fragment ions with *m/z* values of 343 [M–CH_2_O+H]^+^, 312 [M–H_2_O–CO–CH_3_+H]^+^ and 297 [M–H_2_O–CO–CH_2_O+H]^+^. This peak was identified as sinensetin based on a comparison of its MS data with the data from the literature [20, 21]. Peak 11 co-eluted with peak 10 with a retention time of 43.73 min and UV absorption maxima at 270, 295 and 341 nm. In a similar manner to peak 10, MS analysis of peak 11 in the negative ion mode revealed no major peak. However, MS analysis in the positive ion mode revealed a molecular ion peak with an *m/z* value of 343 [M+H]^+^ along with several other ions with *m/z* values of 707 [2M+Na]^+^, 328 [M–CH_3_+H]^+^, 313 [M–CH_2_O+H]^+^ and 285 [M–CH_2_O–CO+H]^+^. The molecular ion peak with an *m/z* value of 343 was subjected to MS^2^ analysis, which resulted in two major daughter ions with *m/z* values of 313 [M–CH_2_O+H]^+^ and 285 [M–CH_2_O–CO+H]^+^. This peak was identified as tetramethyl-*O*-isoscutellarein by comparing its MS data with those from the literature [20].

Peak 12 eluted with a retention time of 46.3 min and gave two UV absorption maxima at 208 and 336 nm. MS analysis in the negative ion mode did not reveal any major peaks, whereas analysis in positive ion mode revealed two protonated molecular ion peaks with *m/z* values of 403 [M+H]^+^ and 373 [M–CH_2_O+H]^+^. The former of these two peaks was subjected to MS^2^ fragmentation analysis, which revealed several fragment ions with *m/z* values of 373 [M–CH_2_O+H]^+^, 388 [M–CH_3_+H]^+^, 355 [M–CH_2_O–H_2_O+H]^+^ and 327 [M–CH_2_O–H_2_O–CO+H]^+^. A comparing of these data with the data from a reference compound and the literature [21] revealed that this peak was hexamethoxyflavone.

Peak 13 eluted with a retention time of 47.1 min and gave a UV absorption maximum of 333 nm. MS analysis in the negative ion mode revealed no obvious peak, whereas analysis in the positive ion mode revealed a protonated molecular ion with an *m/z* value of 403 [M+H]^+^, as well as two other peaks with *m/z* values of 388 [M–CH_3_+H]^+^ and 373 [M–CH_2_O+H]^+^. MS^2^ fragmentation analysis of the protonated molecular ion peak with an *m/z* value of 403 revealed several daughter peaks with *m/z* values of 373 [M–CH_2_O+H]^+^, 388 [M–CH_3_+H]^+^, 343 [M–2CH_2_O+H]^+^, 313 [M–3CH_2_O+H]^+^, 283 [M–4CH_2_O+H]^+^, 239 [M–C_10_H_12_O_2_+H]^+^ and 211 [M–C_10_H_12_O_2_–CO+H]^+^. A comparison of these data with those obtained from a reference compound and the literature [7, 20, 22] revealed that this peak was nobiletin.

Peak 14 eluted with a retention time of 47.7 min and gave two UV absorption maxima at 266 and 322 nm. MS analysis in the negative ion mode did not reveal any major peak, whereas analysis in the positive ion mode gave several peaks with *m/z* values of 343 [M+H]^+^, 365 [M+Na]^+^, 328 [M–CH_3_+H]^+^ and 313 [M–CH_2_O+H]^+^. The peak with an *m/z* value of 343 [M+H]^+^ was subjected to MS^2^ fragmentation analysis and gave two daughter ions with *m/z* values of 313 [M–CH_2_O+H]^+^ and 299 [M–CO_2_+H]^+^. This peak was identified as tetramethyl-*O*-scutellarein based on a comparison of its data with those described in the literature [20].

Peak 15 gave a retention time of 50.1 min and two UV absorption maxima at 253 and 343 nm. MS analysis in the negative ion mode revealed no major ions. However, MS analysis in the positive ion mode revealed five peaks with *m/z* values of 433 [M+H]^+^, 455 [M+Na]^+^, 496 [M+Na+CH_3_CN]^+^, 418 [M–CH_3_+H]^+^ and 403 [M–CH_2_O+H]^+^. The peak with an *m/z* value of 433 [M+H]^+^ was subjected to MS^2^ analysis, resulting in three major daughter ions with *m/z* values of 403 [M–CH_2_O+H]^+^, 373 [M–2CH_2_O+H]^+^ and 345 [M–2CH_2_O–CO+H]^+^. This peak was tentatively identified as 3,5,6,7,8,3’,4′-heptamethoxyflavone based on a comparison with data from the literature [7, 20].

Peak 16 eluted with a retention time of 51.8 min and two UV absorption maxima at 270 and 323 nm. MS analysis in the negative ion mode revealed no major peaks, whereas analysis in the positive ion mode revealed three major peaks with *m/z* values of 373 [M+H]^+^, 395 [M+Na]^+^ and 358 [M–CH_3_+H]^+^. The protonated molecular ion with an *m/z* value of 373 [M+H]^+^ was subjected to MS^2^ fragmentation analysis, which resulted in three daughter peaks with *m/z* values of 343 [M–CH_2_O+H]^+^, 283 [M–3CH_2_O+H]^+^ and 312 [M–CH_3_–H_2_O–CO+H]^+^. This peak was identified as tangeretin based on a comparison of these data with those reported in the literature [7, 20, 22], as well as the data obtained from the reference compounds.

Peak 17 gave a retention time of 56.5 min with four UV absorption maxima at 203, 254, 282 and 343 nm. No major peaks were observed by MS analysis in the negative ion mode, whereas analysis in the positive ion mode revealed four peaks with *m/z* values of 389 [M+H]^+^, 411 [M+Na]^+^, 374 [M–CH_3_+H]^+^ and 359 [M–CH_2_O+H]^+^. The peak with an *m/z* value of 389 [M+H]^+^ was subjected to MS^2^ fragmentation analysis, resulting in two daughter ions with *m/z* values of 359 [M–CH_2_O+H]^+^ and 374 [M–CH_3_+H]^+^. This peak was tentatively identified as 5-hydroxy-6,7,8,3′,4′-pentamethoxyflavone based on a comparison of these data with the data from the literature [7].

Peak 1 eluted with a retention time of 15.2 min and a UV absorption maximum of 283 nm. Peak 7 had a retention time of 36.5 min and three UV absorption maxima at 210, 257 and 342 nm. However, neither of these two peaks gave any major ions when they were analyzed by MS in the negative and positive ionization modes.

Peak 3 had a retention time of 19.2 min with two UV absorption maxima at 253 and 347 nm. MS analysis in the negative ion mode revealed a deprotonated molecular ion peak with an *m/z* value of 607 [M–H]^–^. The subsequent fragmentation of this peak by MS^2^ analysis resulted in a daughter peak with an *m/z* value of 299 [M-Rutinosyl–H]^–^. MS analysis in the positive ion mode resulted in four major peaks with *m/z* values of 609 [M+H]^+^, 631 [M+Na]^+^, 463 [M-Rhamnosyl+H]^+^ and 301 [M-Rutinosyl+H]^+^. MS^2^ fragmentation analysis of the first of these four peaks resulted in a daughter ion with an *m/z* value of 301 [M-Rutinosyl+H]^+^.

Peak 6 eluted with a retention time of 32.4 min and two UV absorption maxima at 254 and 347 nm. Analysis in negative ion mode revealed two peaks with *m/z* values of 732 [M–H]^–^ and 695 [M–CO–H]^–^. The subsequent fragmentation of the former of these two peaks by MS^2^ analysis gave a daughter ion with an *m/z* value of 417 [M-306–H]^–^. MS analysis in the positive ion mode resulted in two peaks with *m/z* values of 725 [M+H]^+^ and 747 [M+Na]^+^. The MS^2^ fragmentation of the former of these two peaks resulted in a daughter peak with an *m/z* value of 419 [M-306+H]^+^.

Peak 8 had a retention time of 40.3 min and gave three UV absorption maxima at 203, 269 and 345 nm. MS analysis in the negative ion mode did not reveal any major peaks, whereas analysis in the positive ion mode gave four peaks with *m/z* values of 373 [M+H]^+^, 395 [M+Na]^+^, 358 [M–CH_3_+H]^+^ and 343 [M–CH_2_O+H]^+^. The first of these four peaks was subjected to MS^2^ analysis, which resulted in a major daughter peak with an *m/z* value of 343 [M–CH_2_O+H]^+^.

Peak 9 had a retention time of 42.2 min with four UV absorption maxima at 206, 253, 270 and 355 nm. MS analysis in the negative ionization mode revealed major peaks whereas analysis in the positive ion mode revealed four peaks with *m/z* values of 403 [M+H]^+^, 425 [M+Na]^+^, 388 [M–CH_3_+H]^+^ and 373 [M–CH_2_O+H]^+^. The peak observed with an *m/z* value of 403 [M+H]^+^ was subjected to MS^2^ fragmentation analysis, resulting in a daughter peak with an *m/z* value of 373 [M–CH_2_O+H]^+^.

Despite of best efforts, we were unable to identify peaks 1, 3, 6–9.

### Fingerprinting and chemometrics

In this study, all the CER samples were compared with the corresponding reference samples (generated from all the CER samples) based on their calculated similarity values (Table 6). We also performed PCA based on the contents of three specific analytes, as well as the PA/W values of the remaining 14 characteristic peaks. This operation can be thought of as revealing the internal structure of the data in a way that explains the variance of the samples.

**Table 6:** Similarity values of each sample compared to the reference fingerprint generated

Sample no.	Similarity			
	Correlation coefficient		Angle cosin	
	Mean^a^	Median^b^	Mean	Median
L-CER-01	0.2414	0.1666	0.4666	0.4017
L-CER-02	0.4443	0.5450	0.5903	0.6573
L-CER-03	0.4422	0.5300	0.6050	0.6599
L-CER-04	0.4327	0.5146	0.6124	0.6602
CER-01	0.8866	0.7544	0.9126	0.8247
CER-02	0.8873	0.7536	0.9140	0.8251
CER-03	0.8856	0.7535	0.9116	0.8238
CER-04	0.8853	0.7562	0.9116	0.8257
CER-05	0.7290	0.8199	0.8180	0.8767
CER-06	0.7193	0.7427	0.7929	0.8098

^a^The reference fingerprint was generated with mean values of the samples

^b^The reference fingerprint was generated with median vales of the samples

**Fig. 2:**
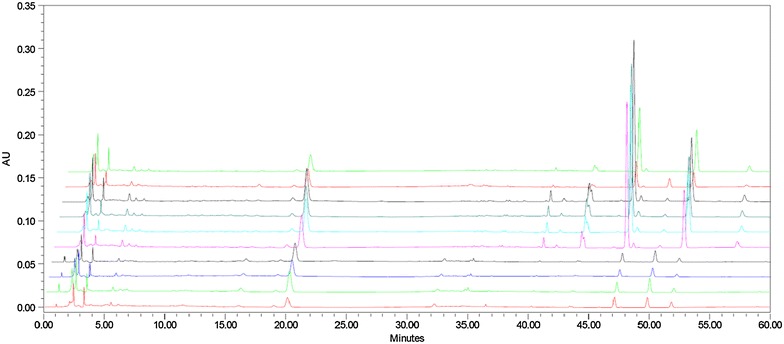
Overlapped chromatographic fingerprints of the testes CER samples

Fingerprinting analysis was conducted using the 17 characteristic peaks shown in the overlapped chromatograms (Fig. 2). These peaks were used as variables to calculate the similarity values. The results of this process showed that the similarity values of the L-CER samples (0.24–0.66) were lower than those of the CER samples (0.72–0.91), indicating that the chemical profiles of the CER samples were similar to that of the reference fingerprint. Based on the contents and PA/W values of the different samples, we determined that the hesperidin, nobiletin and tangeretin contents of the L-CER samples were lower than those of the CER samples. Furthermore, the CER samples contained 10–30 times as much nobiletin and tangeretin as the L-CER samples. Peaks 1, 6 and 7 were not detected in most of the CER samples, however, they were found to be particularly prominent in the L-CER samples. None of the L-CER samples contained a signal corresponding to peak 17 (5-hydroxy-6,7,8,3′,4′-pentamethoxyflavone), which was found in all the CER samples. Although all of the L-CER samples purchased in pharmacies in Hong Kong were actually imported from mainland China, they all showed considerable differences in the chemical profiles. For example, the hesperidin contents of the CER samples were around two times higher than those of the L-CER samples. Further work should therefore be conducted to determine whether these differences in the chemical compositions of the samples could lead to differences in their efficacies in clinical practice.

**Fig. 3:**
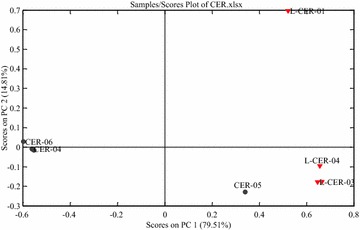
PCA scores plot of CER samples based on the first two components

In this study, the variables for each sample in the PCA consisted of their hesperidin, nobiletin and tangeretin contents, as well as the PA/W values of the remaining 14 characteristic peaks. The resulting data were exported to Excel (Microsoft, Inc., Belleview, WA, USA) to form a two-dimensional matrix (10 samples versus 17 variables), which cumulatively accounted for 94.31 of the total variance, based on which PCA scores plot (Fig. 3) was generated. CER-01, CER-02, CER-03, CER-04 and CER-06 were tightly clustered to the left of the plot, with PC1 scores around −0.6. L-CER-02, L-CER-03 and L-CER-04 were tightly clustered in the lower right of the plot with high PC2 scores. CER-05 was the only CER sample to be found at some distance from the other CER samples, although it was close to the L-CER samples. L-CER-01 was positioned in the upper right of the plot and far away from all of the other samples giving it the highest PC2 score. The loading plot of a variable on a PC generally reflects the extent to which that variable contributes to the PC, and how well the PC accounts for variations in the variable over the data points. The loading results also described the relationships between different variables.

PC1 loadings plot indicated that peak 8, peaks 10 and 11 (sinensetin and tetramethyl-*O*-isoscutellarein), peak 14 (tetramethyl-*O*-scutellarein) and peak 17 (5-hydroxy-6,7,8,3′,4′-pentamethoxyflavone) had made a negative contribution to the positions of the samples tested in PC1. In contrast, peak 1, peak 5 (neohesperidin), peak 6 and peak 15 (3,5,6,7,8,3′,4′-heptamethoxyflavone) had made a positive contribution to PC1. In other words, samples with higher contents or PA/W values of peak 8, peaks 10 and 11 (sinensetin and tetramethyl-*O*-isoscutellarein), peak 14 (tetramethyl-*O*-scutellarein) and peak 17 (5-hydroxy-6,7,8,3′,4′-pentamethoxyflavone) would be positioned to the left of the scores plot. Meanwhile, samples with higher contents or PA/W values of peak 1, peak 5 (neohesperidin), peaks 6 and 15 (3,5,6,7,8,3′,4′-heptamethoxyflavone) would be positioned to the right of the scores plot. In this study, CER-01, CER-02, CER-03, CER-04 and CER-06 had high PA/W values for peak 8, peak 10 + 11 (sinensetin and tetramethyl-*O*-isoscutellarein), peak 14 (tetramethyl-*O*-scutellarein) and peak 17 (5-hydroxy-6,7,8,3′,4′-pentamethoxyflavone), and were consequently positioned to the left of the scores plot. In contrast, all of the L-CER samples had high PA/W values for peak 5 (neohesperidin), peak 6 and peak 15 (3,5,6,7,8,3′,4′-heptamethoxyflavone). The hesperidin, nobiletin and tangeretin contents of CER-05 and the PA/W values of the other characteristic peaks resembled those of the L-CER samples more closely than those of the CER samples.

The loading plot for PC2 showed that peak 1, peak 3, peak 5 (neohesperidin), peak 10 + 11 and peak 15 were having the greatest influence on the samples on the PC2 positions. Peak 5 (neohesperidin) made a positive contribution to the positions, whereas peak 1, peak 3, peak 10 + 11 and peak 15 made a negative contribution to these positions. L-CER-01 had the highest value for peak 5 (neohesperidin) of all of the samples tested in the current study.

### Antioxidant activity of CER samples

#### 2,2-Diphenyl-1-picrylhydrazyl (DPPH) assay

**Fig. 4:**
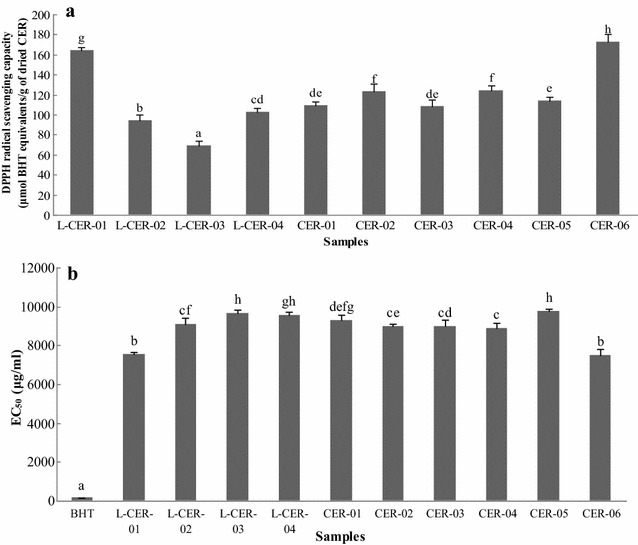
DPPH radical scavenging capacity of CER samples (**a**) and EC_50_ values of BHT and CER samples (**b**). *Different letters* represent significant differences (*P* < 0.05)

The DPPH free radical scavenging activities of the CER samples are shown in Fig. 4a. Each sample was tested at three different concentrations. CER-06 had the highest value at 173.005 μmol butylated hydroxytoluene equivalent (BHTE)/g of dried sample, followed by L-CER-01 at 164.741 μmol BHTE/g of dried sample. L-CER-03 had the lowest value at 68.818 μmol BHTE/g of dried sample, followed by L-CER-02 at 94.372 μmol BHTE/g of dried sample. The EC_50_ values of the samples were calculated and the results are shown in Fig. 4b. Materials with the lowest EC_50_ value exhibited the greatest free radical-scavenging activity. Although the DPPH radical-scavenging activities of all the CER samples tested in the current study were lower than that of BHT, the results showed that the different samples exhibited different radical-scavenging abilities.

#### Ferric ion reducing antioxidant power (FRAP) assay

**Fig. 5:**
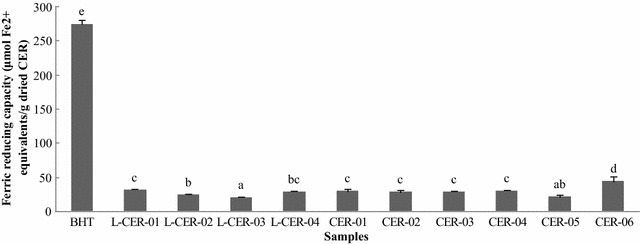
Ferric reducing capacity of CER samples. The values are expressed as mol Fe^2+^ equivalents per gram of dried CER sample (mean ± SD). *Different letters* represent significant differences (*P* < 0.05)

The FRAP values (expressed as Fe^2+^ equivalents) were used to determine the ferric reducing activities of the different CER samples (Fig. 5). The values were between 19.887 (L-CER-03) and 43.890 (CER-06) μmol Fe^2+^ equivalents/g of dried CER sample. The value obtained for BHT was the highest of all of the samples tested at 275.01 μmol Fe^2+^ equivalents/g of BHT. The ferric reducing activities of all of the CER samples were less than that of BHT.

#### Trolox equivalent antioxidant capacity (TEAC) assay

**Fig. 6:**
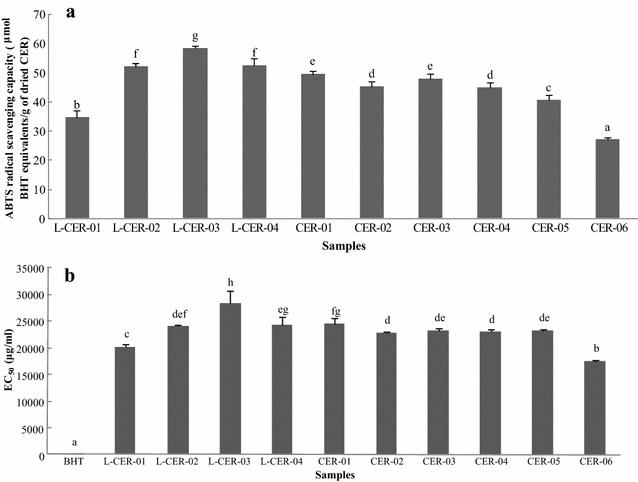
ABTS radical scavenging capacity of CER samples (**a**) and EC_50_ values of BHT and CER samples (**b**). *Different letters* represent significant differences (*P* < 0.05)

The antioxidant activities of the CER samples were determined using the TEAC method, which gave values in the range of 27.120 to 58.391 μmol BHTE/g of dried sample (Fig. 6a). L-CER-03 showed the highest value of all of the samples tested at 58.391 μmol BHTE/g of dried sample, whereas CER-06 gave the lowest value at 27.120 μmol BHTE/g of dried sample. The EC_50_ values were calculated and the results are displayed in Fig. 6b. The differences in the antioxidant activities of the different CER samples were attributed to differences in their chemical compositions.

#### Correlation analysis

Pearson r correlation values were calculated using bivariate correlation analysis because we only evaluated 10 CER samples in the present study, and we were therefore unable to determine whether these samples did or did not obey a Gaussian distribution. The results of this analysis are shown in Table 7, where “–” and “+” have been used to represent negative and positive correlations, respectively. Peaks 03, 04, 08, 10, 11, 13, 14, 16 and 17 positively correlated with the ABTS radical scavenging activity, whereas peaks 01, 06 and 15 negatively correlated with the ABTS radical scavenging activity. None of the peaks correlated with the DPPH free radical scavenging or ferric reducing activity.

**Table 7:** Spearman’s correlation between the characteristic peaks of each CER sample and its antioxidant capacities

Peak no.	Correlation coefficient		
	DPPH free radical scavenging capacity	Ferric reducing capacity	ABTS radical scavenging capacity
Peak 01	−0.575	0.446	−0.760**
Peak 02	−0.290	0.174	−0.406
Peak 03	0.152	−0.224	0.697**
Peak 04	−0.055	0.079	0.709**
Peak 05	0.138	0.450	−0.510
Peak 06	−0.307	0.533	−0.792**
Peak 07	−0.252	0.511	−0.743
Peak 08	0.527	−0.042	0.952**
Peak 09	−0.115	0.394	0.612
Peak 10 + 11	0.503	−0.030	0.976**
Peak 12	−0.018	0.395	0.638*
Peak 13	0.503	−0.030	0.976**
Peak 14	−	−	0.915**
Peak 15	−0.418	0.697	−0.709*
Peak 16	0.503	−0.030	0.976**
Peak 17	0.550	−0.306	0.932**

* *P* < 0.05

** *P* < 0.01

## Conclusion

An HPLC-UV-MS method has been developed and validated after a detailed investigation on extraction of chemical compounds from CER using different solvents and extraction times. None of the peaks correlated with the DPPH free radical scavenging activity or ferric reducing capacity. In contrast, most of the peaks correlated well with the ABTS radical scavenging capacity.

## Additional files

**Additional file 1.** Chromatograms of L-CER-04 extracted using different solvents.

**Additional file 2.** Chromatograms of L-CER-04 extracted using methanol for three times.

**Additional file 3.** Chromatograms of L-CER-04 separated using different columns.
