# Supplementary material for: Chemical compositions, chromatographic fingerprints and antioxidant activities of Citri Exocarpium Rubrum (Juhong)
Source: Chin Med. 2017 Jan 25;12:6. doi: 10.1186/s13020-017-0127-z (PMC5264459; doi:10.1186/s13020-017-0127-z)
Supplement: Supplementary file 1 — Additional file 1. Chromatograms of L-CER-04 extracted using different solvents. [file 13020_2017_127_MOESM1_ESM.doc]

**Extracted using methanol**

Hesperidin

Hesperidin

Nobiletin

Nobiletin

Tangeretin

Tangeretin

**Extracted using 50% methanol**

**✓**

Hesperidin

Nobiletin

Tangeretin

**Extracted using ethanol**

Hesperidin

Nobiletin

Tangeretin

**Extracted using 50% ethanol**
