# Supplementary material for: Chemical compositions, chromatographic fingerprints and antioxidant activities of Citri Exocarpium Rubrum (Juhong)
Source: Chin Med. 2017 Jan 25;12:6. doi: 10.1186/s13020-017-0127-z (PMC5264459; doi:10.1186/s13020-017-0127-z)
Supplement: Supplementary file 2 — Additional file 2. Chromatograms of L-CER-04 extracted using methanol for three times. [file 13020_2017_127_MOESM2_ESM.doc]

**Extracted using methanol for the first time**

Hesperidin

Nobiletin

Tangeretin

**Extracted using methanol for the second time**

Hesperidin

**Extracted using methanol for the third time**

**< 1% of the total**

**No nobiletin and tangeretin**

**peaks were found**
