# Supplementary material for: Chemical compositions, chromatographic fingerprints and antioxidant activities of Citri Exocarpium Rubrum (Juhong)
Source: Chin Med. 2017 Jan 25;12:6. doi: 10.1186/s13020-017-0127-z (PMC5264459; doi:10.1186/s13020-017-0127-z)
Supplement: Supplementary file 3 — Additional file 3. Chromatograms of L-CER-04 separated using different columns. [file 13020_2017_127_MOESM3_ESM.doc]

Alltima C18 (250 mm × 4.6 mm, 5μm)

Unseparated peaks

XBridge Shield RP 18 (250 mm × 4.6 mm, 5μm)

The resolution of the two peaks is less than 1.5

Dikma Inspire C18 (250 mm × 4.6 mm, 5μm)

**✓**
